# Size-dependent electrical transport properties in Co nanocluster-assembled granular films

**DOI:** 10.1038/s41598-017-11983-7

**Published:** 2017-09-15

**Authors:** Q. F. Zhang, X. Z. Wang, L. S. Wang, H. F. Zheng, L. Lin, J. Xie, X. Liu, Y. L. Qiu, Y. Z. Chen, D. L. Peng

**Affiliations:** Department of Materials Science and Engineering, Collaborative Innovation Center of Chemistry for Energy Materials, College of Materials, Xiamen University, Xiamen, 361005 China

## Abstract

A series of Co nanocluster-assembled films with cluster sizes ranging from 4.5 nm to 14.7 nm were prepared by the plasma-gas-condensation method. The size-dependent electrical transport properties were systematically investigated. Both of the longitudinal resistivity (${\rho }_{xx}$) and saturated anomalous Hall resistivity (${\rho }_{xy}^{A}$) continuously increased with the decrease of the cluster sizes (*d*). The ${\rho }_{xx}$ firstly increased and then decreased with increasing the temperature for all samples, which could be well described by involving the thermally fluctuation-induced tunneling (FIT) process and scattering. The tunneling effect was verified to result in the invalidation of classical anomalous Hall effect (AHE) scaling relation. After deducting the contribution from tunneling effect to ${\rho }_{xx}$, the AHE scaling relation between ${\rho }_{xy}^{A}$ and the scattering resistivity (${\rho }_{S}$) by varying the temperature was reconstructed. The value of scaling exponent *γ* increased with increasing Co cluster sizes. The size dependence of *γ* might be qualitatively interpreted by the interface and surface-induced spin flip scattering. We also determined the scaling relation between ${\rho }_{xy}^{A}$ and ${\rho }_{S}$ at 5 K by changing the Co cluster sizes, and a large value of *γ* = 3.6 was obtained which might be ascribed to the surface and interfacial scattering.

## Introduction

Magnetic granular film, as a class of functional materials, is very attractive due to its rich fundamental phenomena and opening a new route for potential novel applications^1–3^. The AHE in ferromagnets, arising from several origins, is one of the most prominent phenomena. For the present theories in homogeneous magnetic materials, it is generally accepted that the skew scattering (*γ* = 1)^4^, side-jump (*γ* = 2)^5^, and intrinsic (*γ* = 2)^6^ mechanisms account for the AHE, which gives an expression of ${\rho }_{xy}^{A}\propto {\rho }_{xx}^{\gamma }$. Therefore, one can parse the microscopic mechanisms of AHE by determining the scaling relation experimentally. However, large number of the theoretical and experimental results revealed that the AHE scaling relation in heterogeneous material was counterintuitive, especially in multilayer film and granular film^7–9^. Song *et al*. studied the AHE in Fe/Cr multilayers and observed a large scaling exponent (*γ* = 2.6) which resulted from the interfacial scattering^8^. Guo *et al*. discovered a large scaling exponent in Co/Pd multilayer films (*γ* = 5.7) which was also ascribed to the interfacial scattering^10^. In addition, Zhang *et al*. observed a large enhancement of ${\rho }_{xy}^{A}$ in Co/Pt multilayers by inserting the MgO/CoO hybrid bilayers^11^. Afterwards, Guo *et al*. also obtained a large enhancement of ${\rho }_{xy}^{A}$ in Co/Pd multilayers by modifying the interfacial structures^12^. These results gave a strong indication that the interfacial scattering could significantly enhance the AHE. The AHE in granular films also have received extensive attention because of their abundant surface/interface and controllable microstructure^13–15^. The microstructure in granular films can be effectively adjusted by governing the experiment parameters. Xiong *et al*. studied the size dependence of ${\rho }_{xy}^{A}$ and ${\rho }_{xx}$ in Co-Ag granular films by changing the annealing temperature, and the scaling exponent of *γ* = 3.7 was obtained at 4.2 K^9^. They also found that the ${\rho }_{xy}^{A}$ increased with the decrease of Co grain sizes. The size dependence of ${\rho }_{xy}^{A}$ was also observed in Co-Cu granular films^16^. These experimental observations implied that the grain size had a crucial effect on AHE. Nevertheless, the annealing affected the microstructure of the granular system by a complicated way^17^. In this case, it was difficult to accurately demarcate the contribution of grain size, interface and interparticle distance to AHE. In the aspects of theoretical research, Granovsky *et al*.^18^ and Vedyaev *et al*.^19^ also suggested that the decrease of the grain sizes would effectively improve the ${\rho }_{xy}^{A}$ and the value of *γ* displayed obvious size-dependent in granular films. Unfortunately, the experimental evidence is still very scarce. The most critical factor is that it is difficult to individually control the grain size and interparticle distance by conventional preparation techniques. In this case, the size-dependent AHE remains an open question. Consequently, it is necessary and interesting to develop a more effective preparation method and study the size-dependent AHE in uniform single phase magnetic granular system.

In this paper, the uniform Co nanocluster-assembled granular films with different Co cluster sizes by plasma-gas-condensation (PGC)-type cluster beam deposition apparatus. With this method, we successfully realized the individual control of cluster size. And then we systematically studied the Co cluster sizes dependence of ${\rho }_{xx}$ and ${\rho }_{xy}^{A}$ in uniform Co nanocluster-assembled films. Both of the ${\rho }_{xx}$ and ${\rho }_{xy}^{A}$ increased with the decrease of cluster sizes which could be attributed to the increase of surface and interfacial scattering. Furthermore, the size-dependent *γ* were investigated and corresponding physical mechanisms were discussed. Our results provide unequivocal experimental evidences for the effect of cluster size on the AHE.

## Results and Discussion

**Figure 1:**
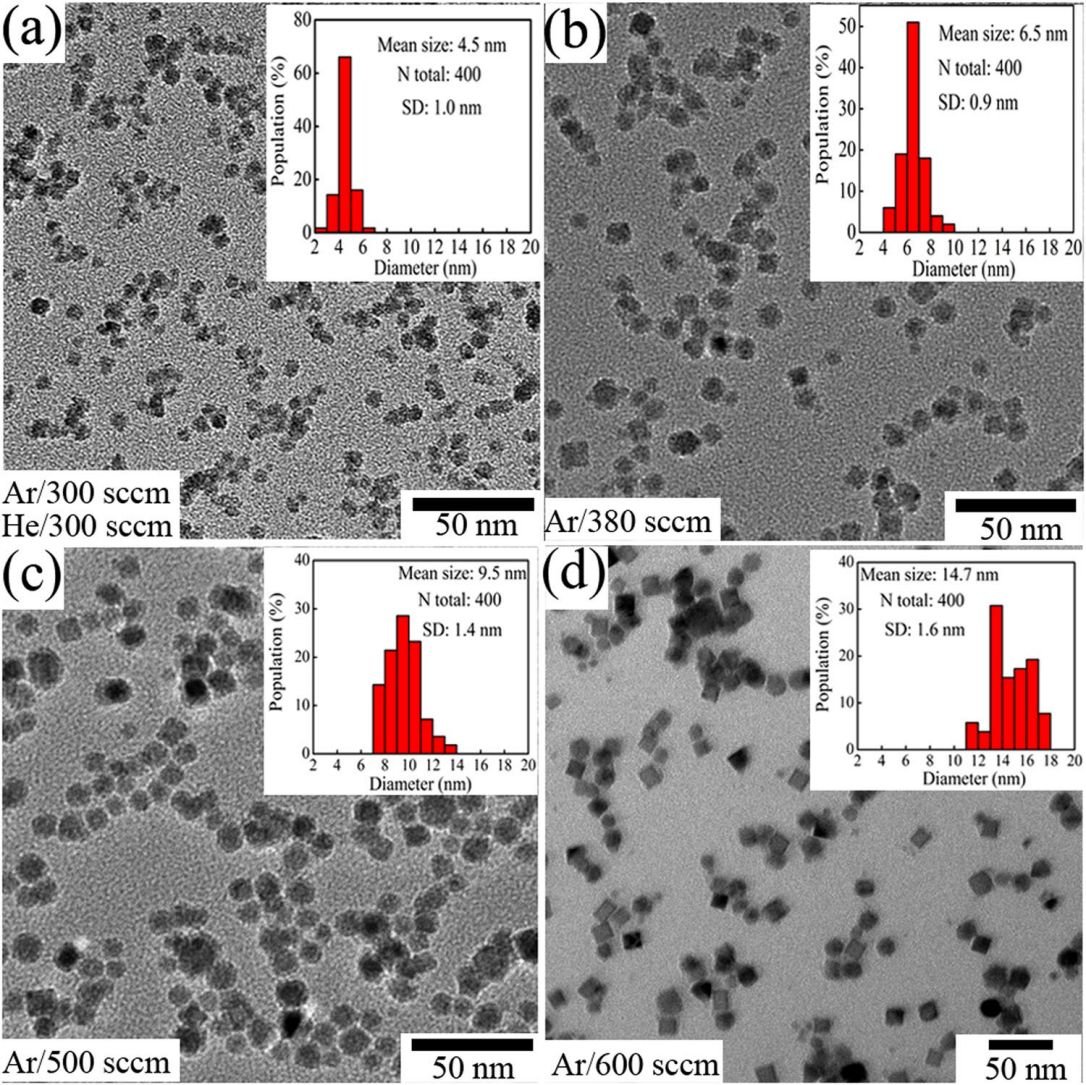
(**a**–**d**) Low-magnification TEM images of Co nanoclusters with different size. The inset in (**a**–**d**) are the corresponding size distribution.

The Low-magnification TEM images and the corresponding size distribution of the Co nanoclusters are displayed in Fig. 1. The statistical results showed that the average size of clusters decreased from 14.7 nm to 6.5 nm as the Ar flow rate reduced from 600 sccm to 380 sccm, and further decreased to 4.5 nm when the 300 sccm He and 300 sccm Ar were simultaneously injected into deposition chamber [Fig. 1(a)]. The narrow size distribution meant that the size of Co clusters for all samples were very uniform. Additionally, the Co clusters were almost cubic geometry at *d* = 14.7 nm while gradually turned into spherical-like with the decrease of Co cluster sizes due to the high-surface-energy and small size effect. Hence, the size and geometry of Co clusters could be adjusted by tuning the Ar and He flow rates.

**Figure 2:**
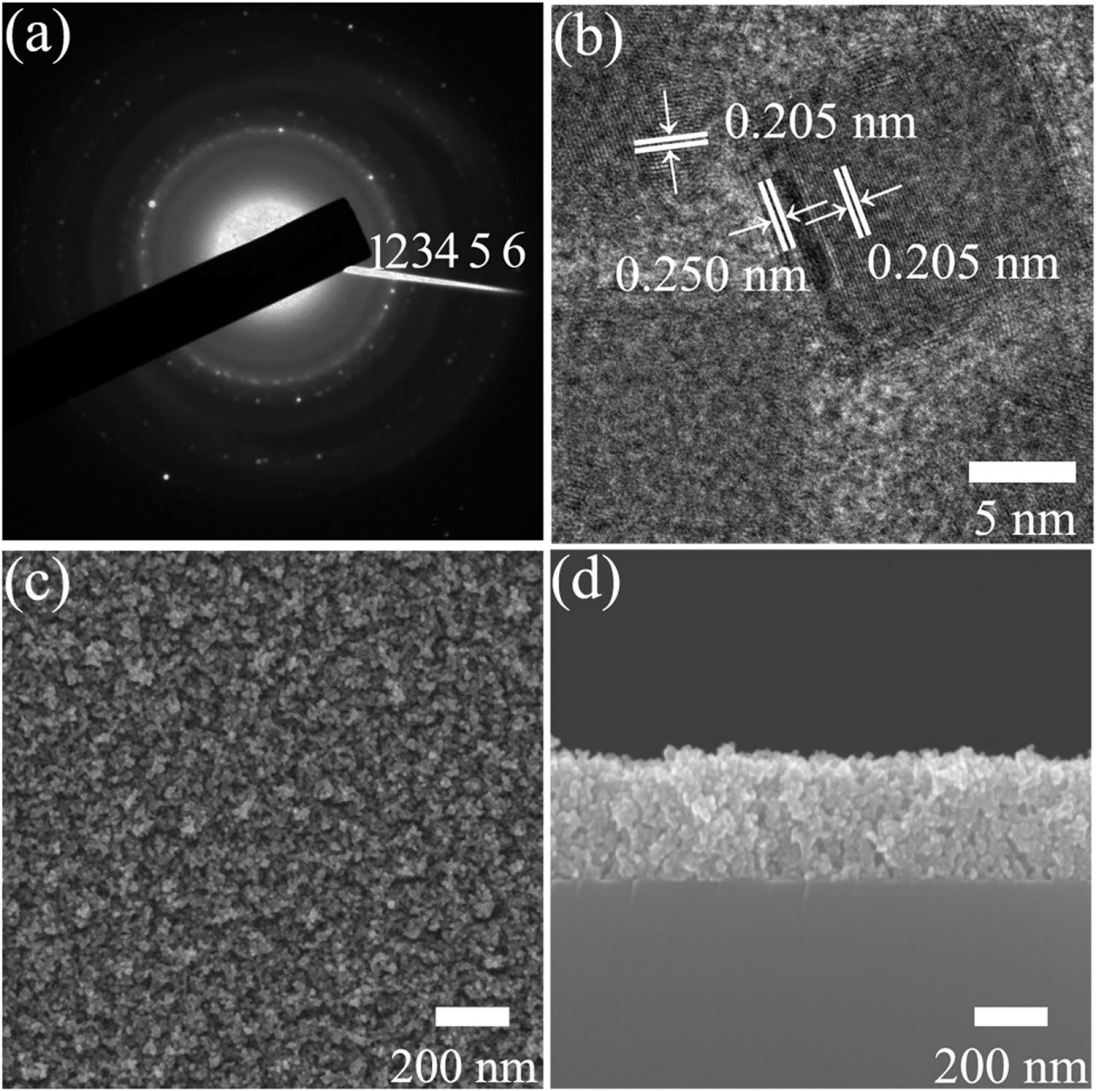
(**a**) SAED pattern, (**b**) high-resolution TEM image, (**c**) plan-view and (**d**) cross-sectional SEM images of the Co nanocluster-assembled film for *d* = 14.7 nm.

Figure 2(a) displays the SAED pattern of Co nanocluster-assembled film for *d* = 14.7 nm. The diffraction rings 2, 3, 5 and 6 corresponded to the {111}, {200}, {220} and {311} planes of metastable fcc Co. The presence of the diffraction rings 1 and 4 were attributed to {111} and {220} planes of fcc CoO, which was possibly originated from slight oxidization when the samples were exposed to the ambient atmosphere. The high-resolution TEM observation of the Co clusters is exhibited in Fig. 2(b). The lattice fringe of 0.205 nm belonged to {111} interplanar spacing of metastable fcc Co. While the lattice fringes of 0.250 nm corresponded to {111} interplanar spacing of fcc CoO, which agreed well with the observation in Fig. 2(a). The adjacent Co clusters connected with each other, forming three-dimensional (3D) effective conductive paths. Figure 2(c-d) show the planar-view and cross-sectional SEM images of the Co cluster-assembled film for *d* = 14.7 nm. These results demonstrated that a porous structure and the randomly stacking of the Co cluster were obtained in granular films.

**Figure 3:**
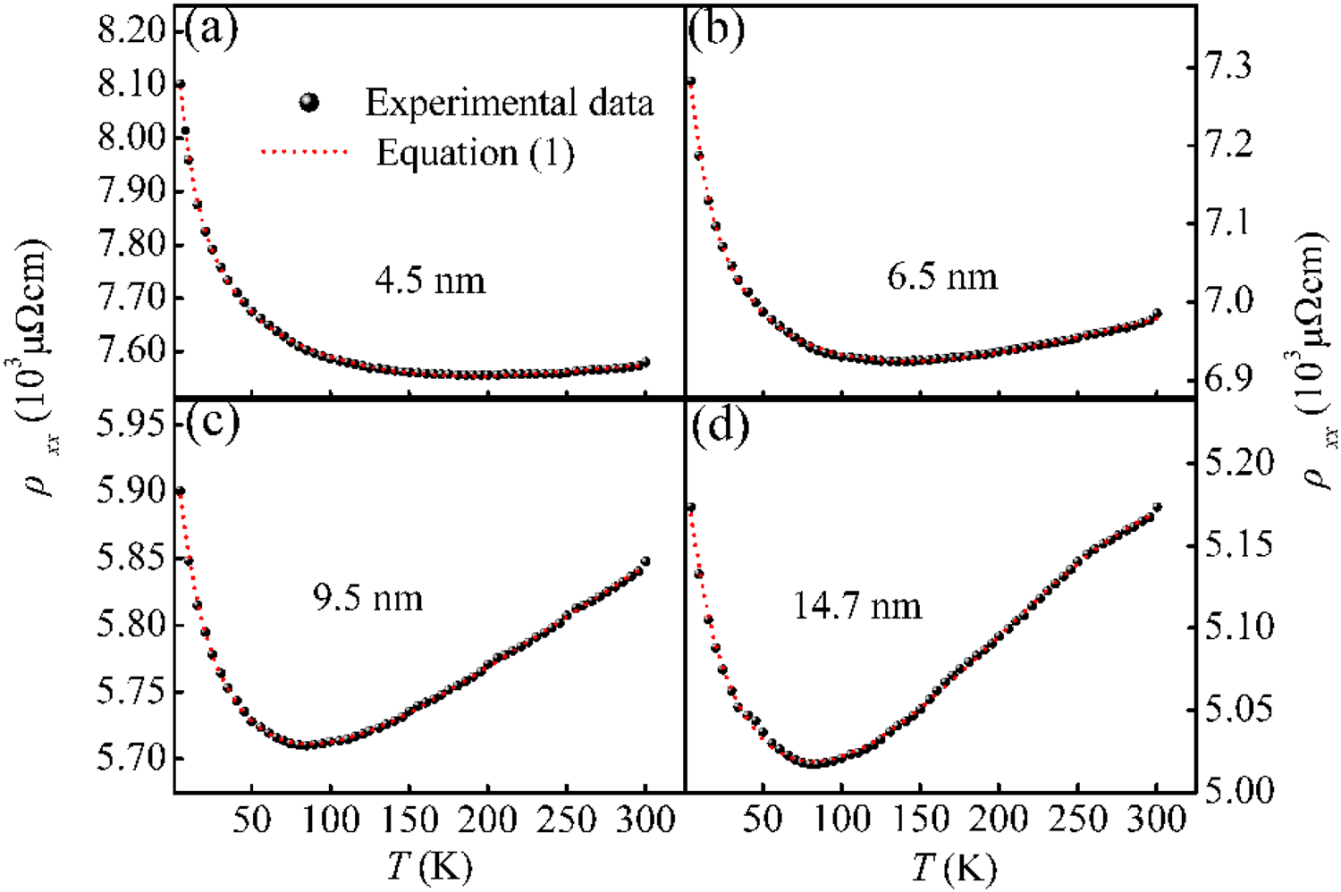
${\rho }_{xx}$ − *T* curves for Co nanocluster-assembled films with different Co cluster sizes. The symbols are the experimental data. The short dash curves are the theoretical forecasting by Eq. (1).

Figure 3 plots the variation in ${\rho }_{xx}$ with the temperature for the four representative samples. In all cases, the ${\rho }_{xx}$ first decreased and then increased with the increase of temperature, resulting in a minimum value at a certain temperature (${T}_{\min }$). In the high temperature region ($T > {T}_{\min }$), all samples exhibited a positive temperature coefficient of resistance (TCR). Nevertheless, a negative TCR was observed in the low temperature range ($T < {T}_{\min }$). It was worth mentioning that the atom distribution at the Co clusters surface and interface was highly disorder and slightly oxidized [Fig. 2(a-b)]. In this case, the interface between adjacent Co clusters could be considered as mesoscopic tunnel junctions and the barrier height was low enough. Under the circumstances, the negative TCR observed in our samples could be attributed to tunneling effect suggested by FIT process^20, 21^. As can be seen from Fig. 3(d), the value of ${\rho }_{xx}$ for *d* = 14.7 nm at 300 K was far larger than the reported value in Co epitaxial thin films^22^, which was attributed to the strong surface/interfacial scattering and tunneling effect in our sample. Guo *et al*. studied the electrical transport properties in polycrystalline Ni films and suggested that the total resistivity could be written as the superposition of tunneling effect and scattering^23^. Hence, the temperature-dependent ${\rho }_{xx}$ in our samples might also be given as:

1 $${\rho }_{xx}={\rho }_{0}\exp (\frac{{T}_{1}}{T+{T}_{0}})+B{T}^{2}+C{T}^{3}+D{T}^{5}.$$

Here, the first term represents the resistivity dominated by FIT process and the residual terms are the contribution from temperature-dependent scattering^21, 24–26^. The *T* ^2^ term includes the contribution from magnetic scattering, surface-induced scattering, and electron-electron scattering. The *T* ^3^ and *T* ^5^ terms are mainly ascribed to the contribution from phonon scattering in the framework of the Bloch-Wilson and Bloch-Gr€uneisen formula, respectively^21^. The coefficient of *B*, *C*, *D* and ${\rho }_{0}$ are constant. ${T}_{1}$ and ${T}_{0}$ are characteristic parameters depending on mesoscopic tunnel junctions, which can be further expressed as^20^:

2 $${T}_{1}=\frac{8{\varepsilon }_{0}A{V}_{0}^{2}}{{e}^{2}{k}_{B}\omega },$$

and

3 $${T}_{0}=\frac{16{\varepsilon }_{0}\hslash A{V}_{0}^{3/2}}{\pi {e}^{2}{k}_{B}{\omega }^{2}\sqrt{2m}}.$$

Here, *ω* is the junction width, *A* is the junction area, and ${V}_{0}$ is the barrier height (See Supplementary Fig. S1). *m* represents the electronic mass, *ε*_0_ is the permittivity of vacuum and *ћ* is reduced Planck’s constant. As can be seen from Fig. 3, the experimental results for all samples agreed well with the theoretically predicted by Eq. (1). This gave a clear indication that the ${\rho }_{xx}$ originated form the superposition of tunneling effect and scattering, and scattering dominated the ${\rho }_{xx}$ in the high temperature range, while FIT process gradually became important with the decrease of temperature. The fitting parameters are displayed in Table 1.

**Table 1:** Data for the fitting parameters attained from Eq. (1).

*d* (nm)	*T*_1_ (K)	*T*_0_ (K)	*T*_1_/*T*_0_
4.5	1.539	15.108	0.102
6.5	1.526	19.111	0.080
9.5	1.566	25.128	0.062
14.7	1.876	37.404	0.050

To further clarify the physical mechanism in Co nanocluster-assembled granular films, we reconstructed the Eqs (2) and (3). The expression of ${T}_{1}/{T}_{0}\propto \omega {V}_{0}^{1/2}$ was obtained. According to the definition in FIT model, the decay length of the tunneling electron wave function inside the barrier is given as $\xi =\hslash /\sqrt{2m{V}_{0}}$. Hence, the ${T}_{1}/{T}_{0}$ can be further written as ${T}_{1}/{T}_{0}\propto \omega /\xi$. This signifies that a lower value of ${T}_{1}/{T}_{0}$ corresponds to a higher electron tunneling probability. As can be seen from Table 1, the value of ${T}_{1}/{T}_{0}$ decreased with increasing the Co cluster sizes, suggesting a higher electron tunneling probability between adjacent Co clusters. Such a behavior revealed that the contribution from tunneling effect decreased with increasing Co cluster sizes. Meanwhile, a higher electron tunneling probability indicated a lower barrier height at interface between adjacent Co clusters. This scenario revealed that the atom distribution disorder at interface decreased with the increase of Co cluster sizes. On the other hand, the number of Co clusters in unit volume deceased with the increasing Co cluster sizes. Hence, the amount of the interface and surface in in unit volume also deceased with the increase of Co cluster sizes, suggesting the decrease of interfacial and surface scattering. Hence, the ${\rho }_{xx}$ decreased monotonously with the increase of Co cluster sizes might mainly result from the less contribution from FIT process, interfacial and surface scattering.

**Figure 4:**
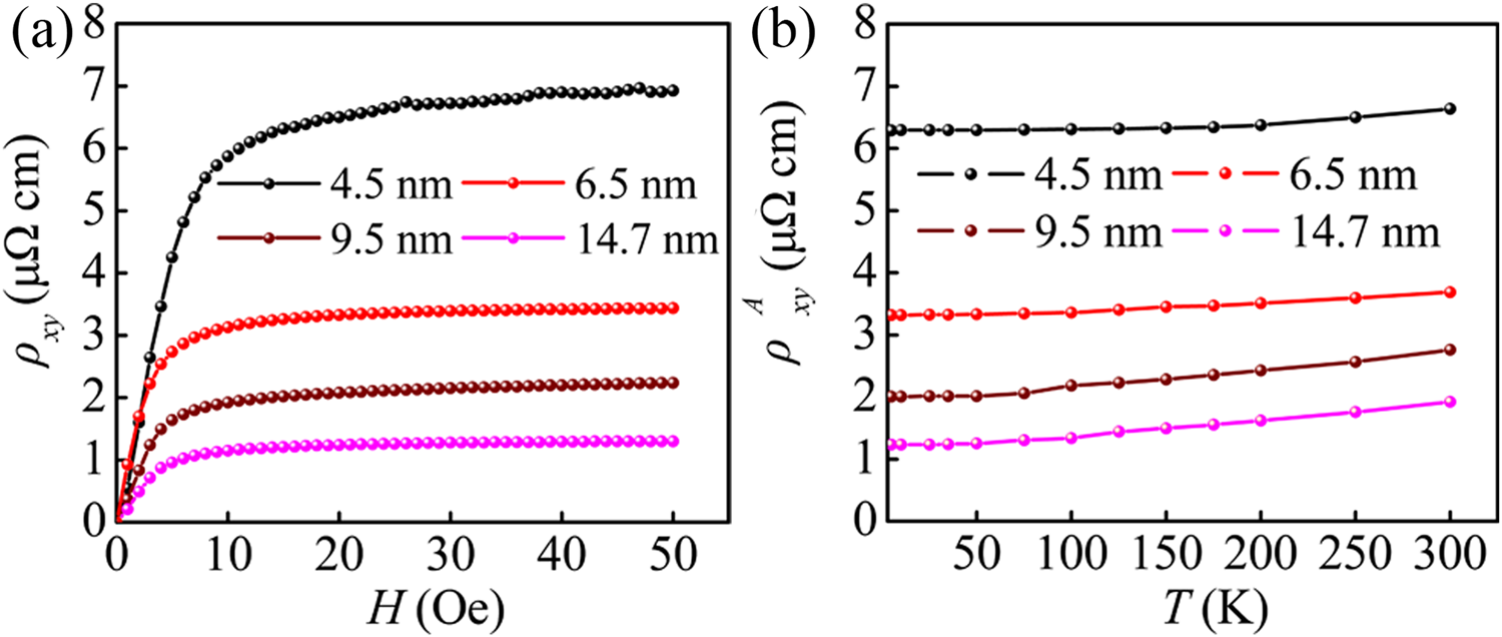
(**a**) The Hall resistivity as a function of field at 5 K, and (**b**) ${\rho }_{xy}^{A}$ − *T* curves for the Co nanocluster-assembled films with different Co cluster sizes.

The field-dependent Hall resistivity ${\rho }_{xy}$ for all the samples at 5 K are plotted in Fig. 4(a). Generally, the Hall resistivity is parameterized by ${\rho }_{xy}={\rho }_{xy}^{O}+{\rho }_{xy}^{A}={R}_{0}H+4\pi {R}_{S}M$ ^27^. *R*_0_, *R*_s_ and *M* are defined as ordinary Hall coefficient, anomalous Hall coefficient and magnetization, respectively. As shown in Fig. 4(a), the ${\rho }_{xy}$ sharply increased at low fields and gradually tended to saturation at high fields. The ${R}_{S}$ could be achieved by mathematical calculation based on the above-mentioned definition formulas of ${\rho }_{xy}$ and the magnetization curves data (See Supplementary Fig. S2). The calculation results of ${R}_{S}$ at 300 K were 1.06 × 10^−8^ (Ω cm)/G, 3.37 × 10^−9^ (Ω cm)/G, 1.79 × 10^−9^ (Ω cm)/G and 1.15 × 10^−9^ (Ω cm)/G for 4.5 nm, 6.5 nm, 9.5 nm and 14.7 nm, respectively. It is worth noting that, the value of ${R}_{S}$ for 4.5 nm Co granular film was almost four orders of magnitude larger than the reported value in blocky single-crystal Co^28^. Such a significant enhancement of Hall coefficient was closely related to large amount of interface and surface. The saturate anomalous Hall resistivity was acquired by using ${\rho }_{xy}^{A}=4\pi {R}_{S}M$ at *H* = 5 T (*M* was measured by Quantum Design physical property measurement system). Referring to this method, the ${\rho }_{xy}^{A}$ with different temperature for the four representative samples are obtained and display in Fig. 4(b). It can be seen form this figure that the value of ${\rho }_{xy}^{A}$ increased continuously with increasing the temperature. Such a behavior was mainly ascribed to surface and interfacial scattering^21^. However, based on the current experimental data, the contribution of intrinsic mechanism could not be ruled out. This behavior was well supported by the results in Co/Pd multilayers^10^. Pay attention to this figure, the ${\rho }_{xy}^{A}$ increased monotonously with the decrease of Co cluster sizes. This was because, as mentioned above, the atom distribution disorder at surface and interface increased with the decrease of Co cluster sizes. It is well known that the AHE was closely related to spin-orbit scattering of conduction electrons at disorder sites in ferromagnet^29, 30^. In the Co nanocluster-assembled films, the amount of surface and interface increased with decreasing Co cluster sizes. These behaviors would effectively enhance the amount of scattering center in the samples. Therefore, the increase of the spin-orbit scattering finally enhanced the ${\rho }_{xy}^{A}$. Such a behavior was supported by the observation in Co/Pd multilayers^12^ and Fe nanocluster-assembled films^31^, which also indicated that surface and interfacial scattering made a significant contribution to improve the AHE. Moreover, this result provided an experimental evidence for the theoretic investigation from Granovsky *et al*.^18^ and Vedyaev *et al*.^19^.

**Figure 5:**
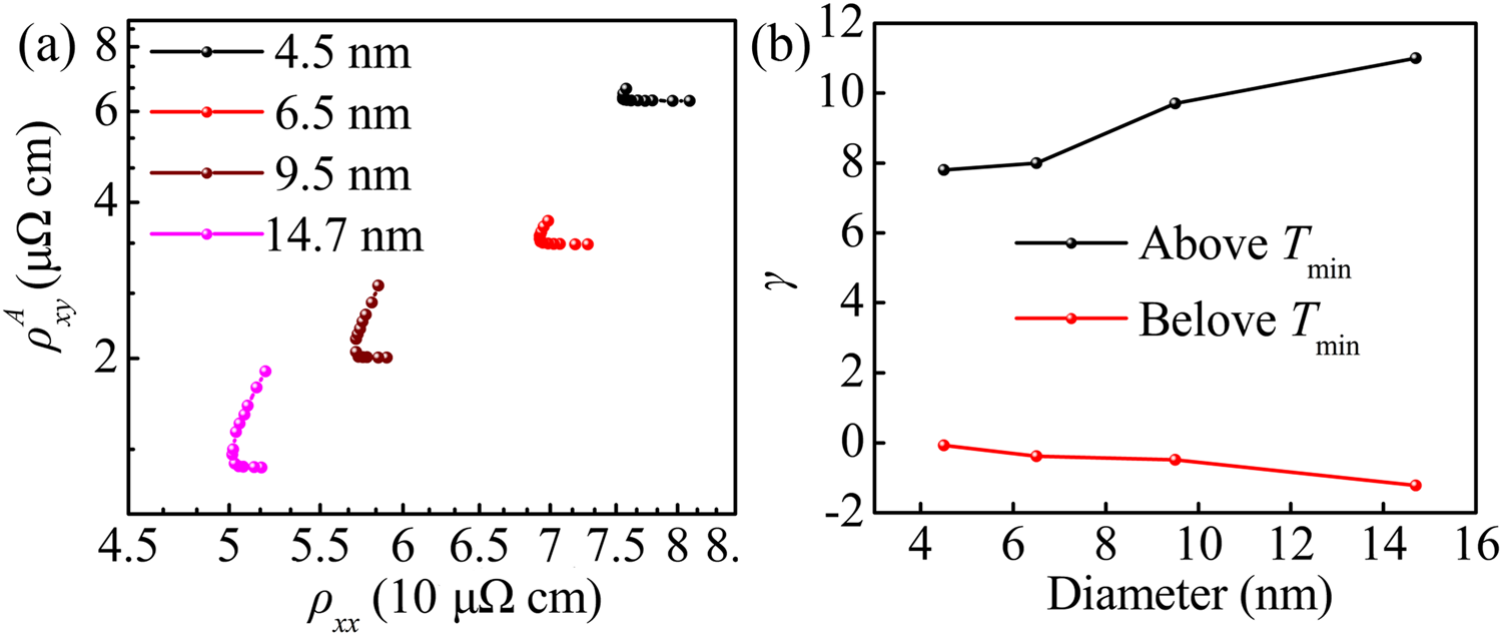
(**a**) The log${\rho }_{xy}^{A}$ − log${\rho }_{xx}$ curves for the Co nanocluster-assembled films with different Co cluster sizes. (**b**) The corresponding scaling exponent of AHE.

To further identify the AHE mechanism in Co nanocluster-assembled films, the ${\rho }_{xy}^{A}$ and ${\rho }_{xx}$ were compiled into a power law of ${\rho }_{xy}^{A}\propto {\rho }_{xx}^{\gamma }$. Figure 5(a) shows the scaling behavior of ${\rho }_{xy}^{A}$ vs ${\rho }_{xx}$ with different Co cluster sizes. It was clear that two distinct regions were observed in log${\rho }_{xy}^{A}$ varying with log${\rho }_{xx}$ for all samples and the demarcation point was the resistivity of ${T}_{\min }$. Fitting the data into straight line, a negative *γ* was observed in the temperature range $T < {T}_{\min }$, while a positive *γ* was found at higher temperature region ($T > {T}_{\min }$). The corresponding *γ* are exhibited in Fig. 5(b). Unfortunately, both of the negative and positive *γ* in our samples could not be explained by classical AHE scaling theory. Moreover, the value of *γ* clearly depended on the Co cluster size. There were some works indicated that the ${\rho }_{xx}$ and ${\rho }_{xy}^{A}$ dominated by different physical mechanisms and the tunneling effect had little effect on AHE^32, 33^. It should be noted that the tunneling effect worked in the whole temperature range. Especially in the temperature range $T < {T}_{\min }$, the ${\rho }_{xx}$ was principally governed by tunneling effect. Therefore, it was no wonder that the classical scaling relation between ${\rho }_{xy}^{A}$ and ${\rho }_{xx}$ was invalid in our systems even in higher temperature range ($T > {T}_{\min }$). To gain insight into the physical mechanisms of AHE in Co nanocluster-assembled films, tunneling effect should be removed from ${\rho }_{xx}$, and the scaling relation between ${\rho }_{xy}^{A}$ and the resistivity only deriving from scattering should be reconsidered.

It was worth mentioning that, as the temperature increased to be in a high limit, the tunneling effect can be neglected. In this case, the resistivity was only originated from scattering. Hence, the Eq. (1) can be rewritten as ${\rho }_{xx(T\to \infty )}={\rho }_{0}+{\rho }_{ST}$. Here, ${\rho }_{ST}$ is the temperature-dependent resistivity, which is given as ${\rho }_{ST}=$ $B{T}^{2}+C{T}^{3}+D{T}^{5}$. ${\rho }_{0}$ is equivalent to residual resistivity which was originated from impurity or imperfection scattering. The scattering resistivity ${\rho }_{S}$ including residual resistivity ${\rho }_{0}$ and temperature-dependent scattering resistivity can be written as:

4 $${\rho }_{S}={\rho }_{0}+{\rho }_{ST}={\rho }_{0}+B{T}^{2}+C{T}^{3}+D{T}^{5}$$

**Figure 6:**
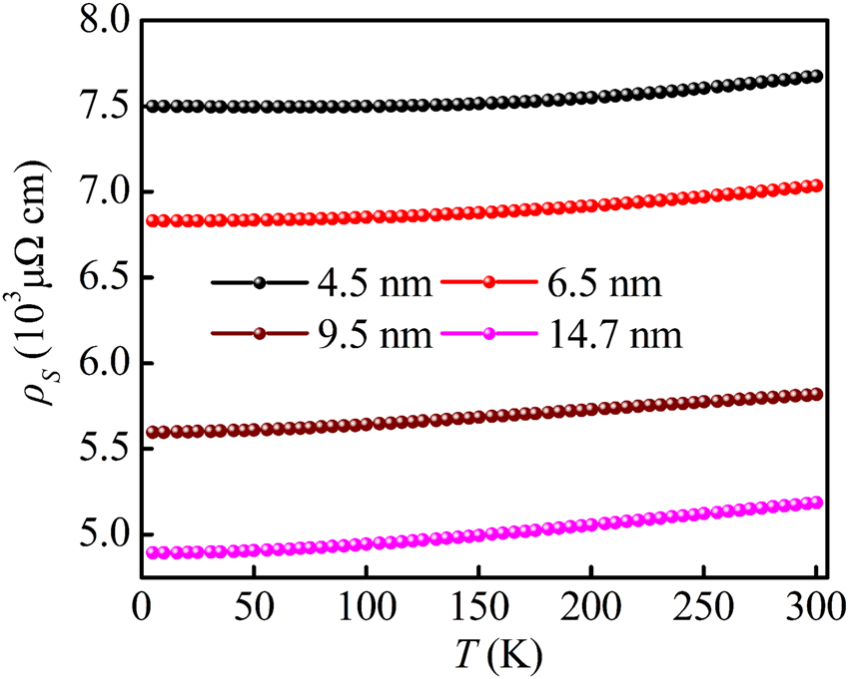
${\rho }_{S}$ − *T* curves for the Co nanocluster-assembled films with different Co cluster sizes.

According to the fitting parameters from Eq. (1), we could effectively evaluate the contribution from ${\rho }_{0}$ and temperature-dependent scattering (${\rho }_{ST}$). In this case, the scattering resistivity with different temperature could be obtained. The ${\rho }_{S}$ − *T* curves with different Co cluster sizes are shown in Fig. 6. Different from the measuring results in Fig. 3, the ${\rho }_{S}$ for all samples continuously increased with increasing the temperature, indicating a behavior of a normal metal conduction characteristic. Moreover, the ${\rho }_{S}$ increased monotonically with decreasing Co cluster sizes. The phenomenon agreed well with the result presented in Fig. 4(b), which was ascribed to the increase of surface and interfacial scattering because of the raise of the amount of scattering center.

**Figure 7:**
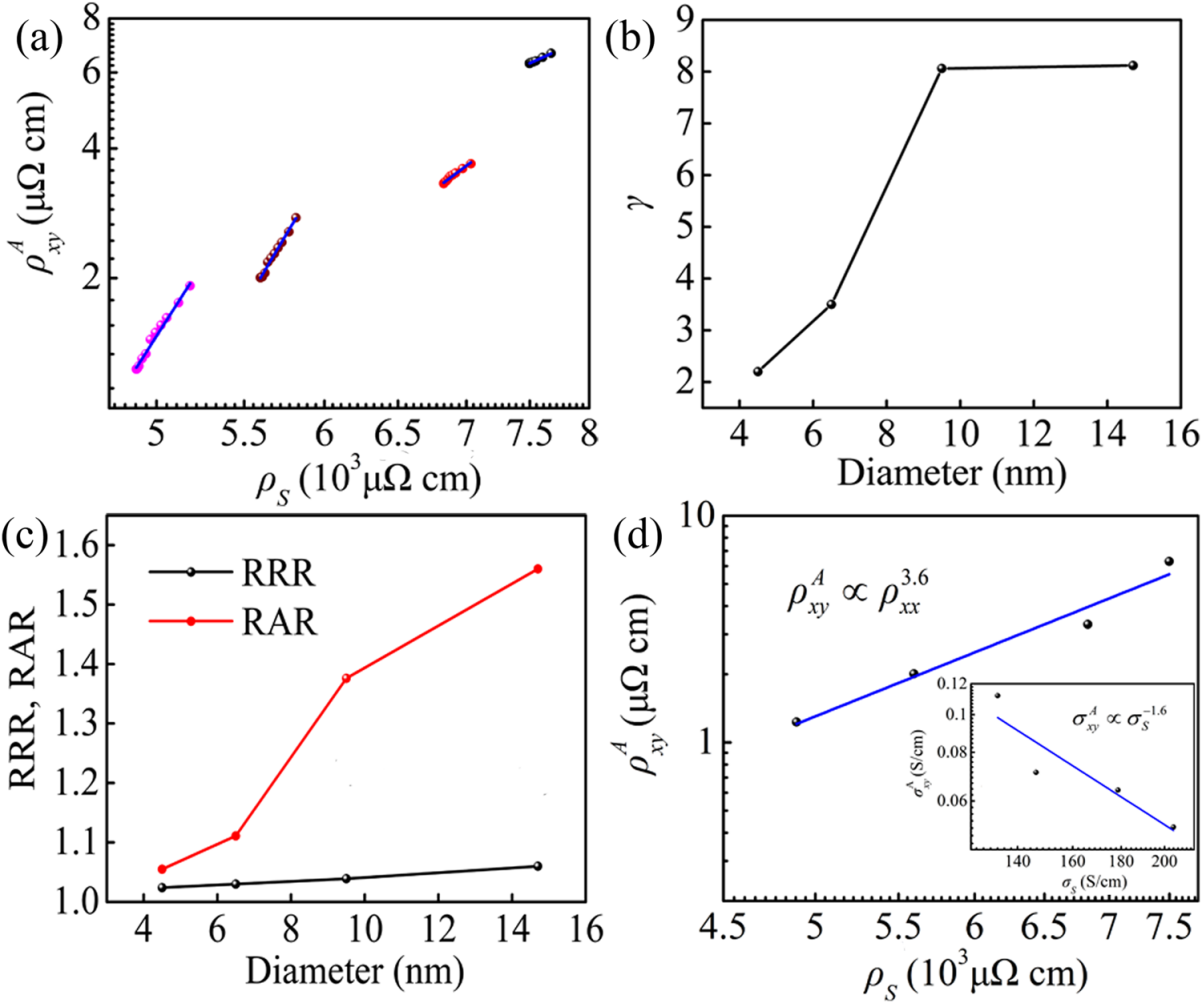
(**a**) The plot of log${\rho }_{xy}^{A}$ − log${\rho }_{S}$ curves for the Co nanocluster-assembled films with different sizes. (**b**) The scaling exponent, (**c**) the RRR and RAR as function of Co cluster sizes. (**d**) The log${\rho }_{xy}^{A}$ − log${\rho }_{S}$ curve at 5 K. The inset shows the scaling of the ${\sigma }_{xy}^{A}$ becomes ${\sigma }_{xx}$.

The AHE scaling between ${\rho }_{xy}^{A}$ and ${\rho }_{S}$ with different Co cluster sizes are exhibited in Fig. 7(a). Remarkably different from the observation in Fig. 5(a), the log${\rho }_{xy}^{A}$ increased monotonously with increasing log${\rho }_{S}$ in the whole temperature range. This observation gave an unambiguous indication that after deducting the contribution from tunneling effect the scaling relation was constructed successfully. By fitting the data to a straight line, the positive scaling exponent for all samples were observed but all of *γ* were larger than 2. The large scaling exponent observed in our samples could be qualitatively attributed to surface and interfacial scattering. The present results agreed with the previously reported in some other heterogeneous systems, such as Fe/Cr multilayers (*γ* = 2.6)^8^, ε-Fe_3_N nanocrystalline films (*γ* = 17.6)^34^ and Fe/Au multilayers (*γ* = 2.7)^35^. More importantly, as shown in Fig. 7(b), the scaling exponent *γ* increased with increasing Co cluster sizes. To some extent, the *γ* could be considered as a quantified criterion to evaluate how fast the ${\rho }_{xy}^{A}$ increased with the raise of ${\rho }_{S}$. In our samples, both of the ${\rho }_{xy}^{A}$ and ${\rho }_{S}$ approximately originated from three parts: bulk scattering, interface scattering and surface scattering^21^. According to the previous report in Co epitaxial thin films^22^, the contribution from bulk scattering was independent on thickness of film. Unlike the Co epitaxial film, there was lager amount of surface and interface in our samples, which would introduce additional scattering center. On the one hand, as mentioned above, the increasing scattering center would effectively increase both of the ${\rho }_{xy}^{A}$ and ${\rho }_{S}$. On the other hand, great scattering strength could cause the spin flip to randomize the spins^10, 36, 37^, which would effectively reduce the spin-dependent scattering ratio but had a limited impact on ${\rho }_{S}$. To gain a deeper understanding of this behavior, the residual resistivity ratio [RRR = ${\rho }_{S}$(300 K)/${\rho }_{S}$(5 K)] of all samples was studied. The RRR is considered as a qualitative method to identify degree of atomic disorder and lattice defect^38^. It should be noted that, at 5 K, the contribution from temperature-dependent scattering could be nearly neglected. In this case, the scattering resistivity was approximately equivalent to residual resistivity [${\rho }_{S}(5{\rm{K}})\approx {\rho }_{0}$]. In analogy with RRR, we defined the residual saturate anomalous Hall resistivity ratio as RAR = ${\rho }_{xy}^{A}$(300 K)/${\rho }_{xy}^{A}$(5 K). The size dependence of RRR and RAR are displayed in Fig. 7(c). Obliviously, the values of RRR decreased with the drop of Co nanocluster sizes, suggesting the improvement of degree of atomic disorder and lattice defect with decreasing cluster size. This conclusion reconfirmed the result mentioned above (${T}_{1}/{T}_{0}$). Furthermore, it was obvious that RAR shown a sharper decrease than RRR with the decrease of Co cluster sizes. This indicated that temperature-dependent ${\rho }_{xy}^{A}$ and ${\rho }_{S}$ were affected by different physical mechanisms, and the spin flip enhanced at high density of interface and surface. As a consequence, the size-dependent *γ* in the Co nanocluster-assembled films might be qualitatively interpreted by the enhancement of interface and surface-induced spin flip to randomize the spins. Such a behavior was in good accord with the experimental investigation in Co/Pd multilayers^10^ and the theoretical research from Granovsky *et al*.^18^ and Vedyaev *et al*.^19^.

Nagaosa *et al*. proposed that the more proper test of the scaling relation was at low temperature by varying the defect concentration where the resistivity was dominated by impurity scattering because the interference from temperature-dependent-scattering could be excluded^27^. Hence, in order to further clarify the physical mechanism of AHE in our samples, we should exclude the effect from temperature-dependent-scattering and tunneling effect and tested the scaling relation between ${\rho }_{S}$ and ${\rho }_{xy}^{A}$ at low temperature. The log${\rho }_{xy}^{A}$ − log${\rho }_{S}$ curve at 5 K is plotted [Fig. 7(d)]. The log${\rho }_{xy}^{A}$ increased monotonically with the increase of log${\rho }_{S}$, which followed a linear behavior. Carrying on the data fitting to the experimental results, the scaling exponent $\gamma =3.6\pm 0.4$ was observed. Therefore, the value of $\gamma =3.6\pm 0.4$ was related to surface and interfacial scattering. Recently, another interesting finding was that Nagaosa *et al*.^27^ proposed the unified theory of the AHE based on a large body of experimental results and theoretical study. The theory predicted that three broad regimes were distinguished as a function of longitudinal conductivity (${\sigma }_{xx}$). (i) ${\sigma }_{xx} > {10}^{6}$ S/cm (high conductivity regime), skew scattering mechanism dominates the Hall transport. (ii) 10^4^ S/cm <${\sigma }_{xx}$< 10^6^ S/cm (intrinsic regime) in which anomalous Hall conductivity ${\sigma }_{xy}^{A}$ becomes ${\sigma }_{xx}$ independent. (iii) ${\sigma }_{xx} < {10}^{4}$ S/cm (bad metal regime), where ${\sigma }_{xy}^{A}\propto {\sigma }_{xx}^{1.6}$ is predicted. The value of the ${\sigma }_{xy}^{A}$ and ${\sigma }_{xx}$ are estimated separately by ${\sigma }_{xy}^{A}\approx {\rho }_{xy}^{A}/{\rho }_{xx}^{2}$ and ${\sigma }_{xx}\approx 1/{\rho }_{xx}$ because of ${\rho }_{xy}^{A} <  < {\rho }_{xx}$_._ As shown in the inset of Fig. 7(d), apparently, all of our samples were in the bad metal regime. Fitting the experimental data into a straight line, ${\sigma }_{xy}^{A}\propto {\sigma }_{S}^{-1.6}$ was obtained at 5 K (${\sigma }_{S}$ represented the longitudinal conductivity originating from scattering), which disagreed with the universal character of the 1.6 scaling relation. This result gave a strong indication that our samples was inconsistent with the unified theory, which was mainly ascribed to the surface and interfacial scattering. The similar phenomenon also could be observed in ε-Fe_3_N nanocrystalline films^34^.

In summary, the uniform Co nanocluster-assembled films with different Co cluster sizes were successfully prepared by the plasma-gas-condensation method. For all samples, the longitudinal resistivity could be very well fitted by the combination of FIT process and scattering in the whole temperature range. Both of ${\rho }_{xx}$ and ${\rho }_{xy}^{A}$ shown a decreasing function of the Co cluster sizes due to the drop of surface and interfacial scattering. The scaling relation of ${\rho }_{xy}^{A}\propto {\rho }_{xx}^{\gamma }$ could be divided into two parts while obeyed the same line relationship between ${\rho }_{xy}^{A}$ and ${\rho }_{S}$ in the whole temperature range, indicating that it was necessary to remove the tunneling effect in establishing the AHE scaling relation. The large scaling exponent (*γ* > 2) observed in our samples could be ascribed to the surface and interfacial scattering and the size-dependent scaling exponent were closely related to the interface and surface-induced spin flip scattering. The large anomalous Hall coefficient [*R*_*s*_ ~ 1.06 × 10^−8^ (Ω cm)/G] observed *d* = 4.5 nm could be attributed to the large amount of surface and interface, which is especially valuable in practical application in Hall device applications.

## Methods

The experimental apparatus of plasma-gas-condensation (PGC)-type cluster deposition system was employed to prepare the samples. The apparatus was simply divided into three regions: a sputtering chamber, a cluster growth room and a deposition chamber^39^. The nucleation and growth of Co clusters occurred mainly in the sputtering chamber. And then Co clusters were extracted twice in the cluster growth room to prevent further growth. Finally, the Co clusters entered into the deposition chamber and softly deposited onto substrate randomly. The DC power of 400 W was used to generate high density metal Co vapor. The argon gas (99.999%) and helium gas (99.999%) with different flow rates (Ar: 600 sccm, Ar: 500 sccm, Ar: 400 sccm, and Ar/He: 300 sccm/300 sccm) were injected continuously into sputtering chamber to control the Co cluster sizes. JEOL JEM-2100 transmission electron microscope (TEM) was used to carry out transmission electron microscopy (TEM) analysis. The morphology and crystalline phase of Co clusters were determined by TEM images and the selected area electron diffraction (SAED) was used to identify crystalline phase. The surface micro-morphology images of Co clusters-assembled films were acquired by SU-70 scanning electron microscope (SEM). The magnetic and electrical properties were performed on Quantum Design physical property measurement system (PPMS-9) with temperatures ranging from 5 to 300 K and the magnetic field sweeping from −5 to 5 T. For each sample, the longitudinal and transverse voltage could be measured simultaneously because of the five contacts in Hall bar. The thickness of cluster-assembled films (~700 nm) were determined by the surface profiler (Alpha-Step D-100).

## Electronic supplementary material

Supplementary Information
