# Supplementary material for: Size-dependent electrical transport properties in Co nanocluster-assembled granular films
Source: Sci Rep. 2017 Sep 15;7:11666. doi: 10.1038/s41598-017-11983-7 (PMC5601485; doi:10.1038/s41598-017-11983-7)
Supplement: Supplementary file 1 — Supplementary Information [file 41598_2017_11983_MOESM1_ESM.pdf]

Size-dependent electrical transport properties in Co nanocluster-assembled granular films

Q. F. Zhang, X. Z. Wang, L. S. Wang,* H. F. Zheng, A. M. Su, L. Lin, J. Xie, X. Liu, Y. L. Qiu, Y. Z. Chen, D. L. Peng*

Department of Materials Science and Engineering, Collaborative Innovation Center of Chemistry for Energy Materials, College of Materials, Xiamen University, Xiamen 361005, China

Supporting Display items

The Co nanocluster-assembled film was constituted by Co clusters separated by an interface. Due to the highly disordered arrangement of atoms at the interface between adjacent Co clusters and partly oxidized Co clusters when the sample was exposed to the ambient atmosphere, the interface could be treated as mesoscopic tunnel junctions with low barrier height (V_0).

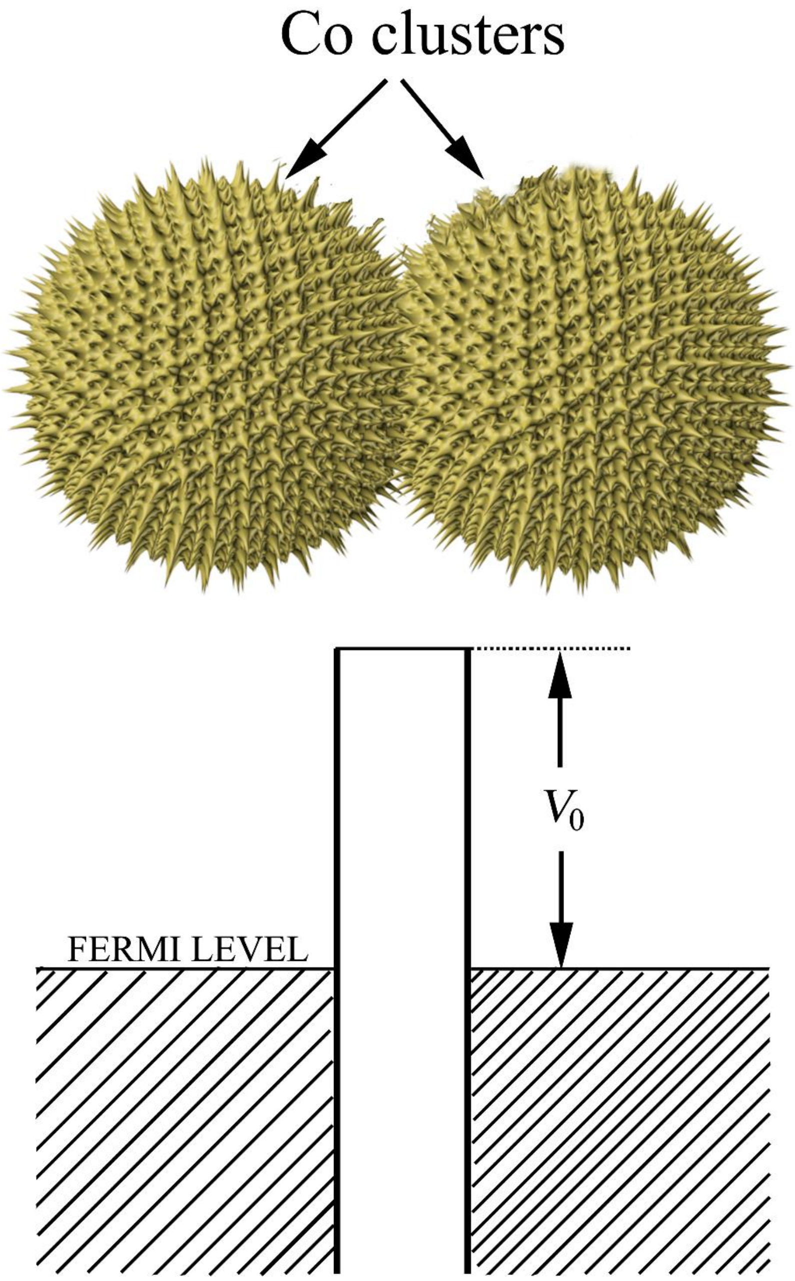

Figure S1. The diagram of the barrier at interface between adjacent Co clusters.

The field-dependent magnetization curves with different Co cluster sizes are displayed in Figure S2. The behavior of the magnetization curves was typical soft ferromagnetic film. Obviously, saturated magnetization (M_S) decreased with decreasing Co cluster sizes, which might be ascribed to the increase of the amount of surface and interface.

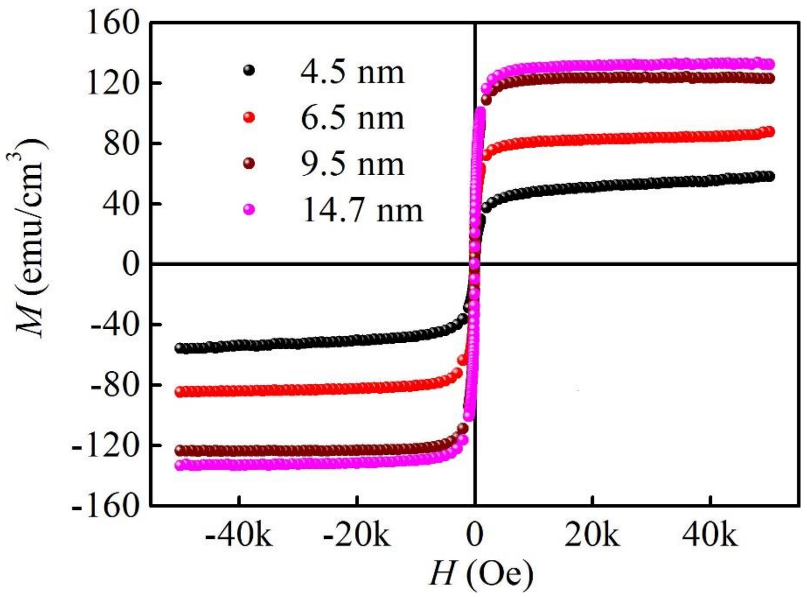

Figure S2. *M-H* curves for the Co cluster-assembled films with different Co cluster sizes.
